# A systematic review of the next‐day effects of heavy alcohol consumption on cognitive performance

**DOI:** 10.1111/add.14404

**Published:** 2018-08-30

**Authors:** Craig Gunn, Marlou Mackus, Chris Griffin, Marcus R. Munafò, Sally Adams

**Affiliations:** ^1^ UK Centre for Tobacco and Alcohol Studies and Department of Psychology University of Bath Bath UK; ^2^ Utrecht Institute for Pharmaceutical Sciences, Department of Psychopharmacology Utrecht University Utrecht the Netherlands; ^3^ UK Centre for Tobacco and Alcohol Studies and School of Experimental Psychology University of Bristol Bristol UK; ^4^ MRC Integrative Epidemiology Unit (IEU) at the University of Bristol Bristol UK

**Keywords:** Alcohol, cognition, driving, hangover, memory, psychomotor, sustained attention

## Abstract

**Background and aims:**

Studies examining the next‐day cognitive effects of heavy alcohol consumption have produced mixed findings, which may reflect inconsistencies in definitions of ‘hangover’. Recent consensus has defined hangover as ‘mental and physical symptoms, experienced the day after a single episode of heavy drinking, starting when blood alcohol concentration (BAC) approaches zero’. In light of this, we aimed to review the literature systematically to evaluate and estimate mean effect sizes of the next‐day effects of heavy alcohol consumption on cognition.

**Methods:**

Embase, PubMed and PsycNET databases were searched between December 2016 and May 2018 using terms based on ‘alcohol’ and ‘hangover’. Studies of experimental designs which reported the next‐day cognitive effects of heavy alcohol consumption in a ‘hangover’ group with BAC < 0.02% were reviewed. A total of 805 articles were identified. Thirty‐nine full‐text articles were screened by two independent reviewers and 19 included in the systematic review; 11 articles provided sufficient data to be included in the meta‐analysis; 1163 participants across 19 studies conducted since 1970 were included in the analysis. Data for study design, hangover severity, BAC at testing and cognitive performance were extracted and effect estimates calculated.

**Results:**

The systematic review suggested that sustained attention and driving abilities were impaired during hangover. Mixed results were observed for: psychomotor skills, short‐ (STM) and long‐term memory (LTM) and divided attention. The meta‐analysis revealed evidence of impairments in STM [*g* = 0.64, 95% confidence interval (CI) = 0.15–1.13], LTM (Hedges’ *g* = 0.59, 95% CI = 0.01–1.17) sustained attention (*g* = 0.47, 95% CI = 0.07–0.87) and psychomotor speed (Hedges’ *g* = 0.66, 95% CI = 0.31–1.00) during alcohol hangover.

**Conclusion:**

The research literature suggests that alcohol hangovers may involve impaired cognitive functions and performance of everyday tasks such as driving.

## Introduction

Research examining the cognitive effects of alcohol hangover have produced conflicting findings. While several studies report impairment in spatial and visual abilities 1, 2, attention 3, 4, 5, 6, 7, memory 6, 8, 9, 10, 11, information processing speed 3, 12, reaction times 8, 9, 12 and intellectual processes 1, 2, others reveal no clear evidence that hangover affects cognition 1, 5, 6, 11, 13, 14, 15, 16, 17, 18, 19, 20, 21, 22. Tasks reflecting work‐place performance have also produced mixed results, with impairments in driving 23, 24, 25, 26, flying 27, 28, 29 and surgical performance 30, 31, 32, but not managerial decisions 33 or problem‐solving in a ship engine 34.

Disagreements in the definition of alcohol hangover may contribute to inconstancies with study designs and measures 35, 36. Some researchers argue that hangover constitutes any next‐day effects following a night of heavy alcohol consumption, and often do not measure blood alcohol concentration (BAC) or hangover at the time of testing. However, some individuals may be hangover‐resistant 37, 38, 39, experiencing no symptoms despite sufficient alcohol to induce hangover. Indeed, the importance of measuring hangover symptoms is highlighted in a recent definition, which received consensus from academics in the field. It states that hangover is a ‘combination of mental and physical symptoms, experienced the day after a single episode of heavy drinking, starting when BAC approaches zero’ 40.

Peak BAC during a night of ‘heavy’ drinking may also contribute to conflicting results 35, 41. To induce a hangover, high amounts of alcohol (> 1 g/kg) are consumed 42, and the higher the amount the more severe are the cognitive impairments 5. Hangovers are studied using either an experimental approach, where an alcohol challenge is administered, or using the naturalistic approach, where participants consume alcohol at a time and place typical for the individual. In experimental studies, hangover may not be induced reliably, as practical and ethical issues could prevent doses > 1 g/kg being administered—again highlighting the need to include measures of hangover in order to validate the hangover condition. Conversely, naturalistic studies have reported alcohol consumption at approximately 1.6 g/kg 9 yet, unlike experimental studies, do not allow for the control of extraneous variables (e.g. food). Although naturalistic and experimental methods may reveal different impairments, it is important to assess convergence of findings across these different methodologies 43, 44.

Previous reviews have highlighted other methodological limitations which contribute to conflicting findings, preventing firm conclusions 36, 41, 45, 46, 47. These include; no BAC measurement at testing, no counterbalance to avoid order effects and poor controls of potentially confounding factors. These reviews excluded studies with BAC > 0 at testing 36, 41, 46. However, alcohol hangover starts when BAC is approaching zero 40, indicating that these reviews may have excluded potentially informative studies. As acute intoxication can produce cognitive effects at BAC > 0.02%, studies which include participants above this threshold cannot disassociate hangover from acute intoxication effects.

The perspective taken here is that BAC should be < 0.02% at testing and hangover symptoms should be measured to validate the hangover condition. However, we acknowledge that, despite mean scores indicating higher hangover severity in hangover conditions, individuals within these groups may not experience hangover symptoms. As separate analysis is not typically reported for those with and without hangover following heavy alcohol consumption, this review should be regarded as examining next‐day effects of heavy alcohol consumption. We acknowledge that hangover has also been explored in animal models; however, the translational value of this work is currently unclear, and so only human studies are included in this review.

To our knowledge, there have been no previous systematic reviews that have estimated mean effect sizes in a meta‐analysis. This review aims to critically evaluate and estimate mean effect sizes to explore the next‐day cognitive effects of heavy alcohol consumption.

## Methods

### Search strategy and inclusion criteria

A literature search was conducted from December 2016–May 2018 to identify studies examining the cognitive effects of alcohol hangover. PubMed, Embase and PsycNET were searched using the strategy ‘alcohol’ OR ‘ethanol’ OR ‘alcohol intoxication’ OR ‘alcohol drinking patterns’ AND ‘hangover’ OR ‘next day effects’. Search terms were adapted for each database and references searched for additional articles. Articles were screened by two independent reviewers and disagreements resolved by discussion in the first instance. If consensus was not reached, a third reviewer was consulted. The inclusion criteria for studies were developed based upon the consensus on hangover research report 48. Only studies that examined healthy human adults (18+ years of age) and contained a no‐hangover control condition were included in the review. Studies had to include a measure which validated the presence of hangover, such as a questionnaire assessing symptoms, and were required to report a BAC < 0.02% at testing. The inclusion criteria were based on a stringent set of criteria for hangover; however it is acknowledged that other approaches may be more inclusive of studies (e.g. including studies which do not include a measure of hangover or BAC at testing).

### Data extraction

Data were extracted from included studies for study design, cognitive tasks, hangover measurement and BAC during hangover. Where possible, quantitative data were extracted and effect estimates calculated 49, 50. Tasks were coded into their corresponding cognitive components 51. Components and their subcategories comprised: attention/vigilance (selective, sustained, divided and vigilance attention), memory [working memory (WM), short‐term memory (STM) and long‐term memory (LTM)] and psychomotor (speed and accuracy).

### Data analysis

All meta‐analyses were performed using RevMan 52. Hedges’ *g* effect size estimates were calculated 18, 19 for each outcome. For those studies with multiple outcomes in each category of cognition, effect sizes were averaged so that no study carried undue weight in determining overall effect. The weight given to each study was the inverse of the variance of the effect size, thus larger studies with smaller standard errors were given more weight.

## Results

### Identification of studies

Agreement between reviewers was 95% with two ‘disagreements’ which were resolved through discussion, without the need to consult a third reviewer. In one case, upon both reviewers revisiting the paper, it was clear that the paper did not measure hangover. In the other case, inclusion criteria for one study were reported across two papers. The reviewers agreed that the inclusion criteria were met by collating data from both papers.

**Figure 1:**
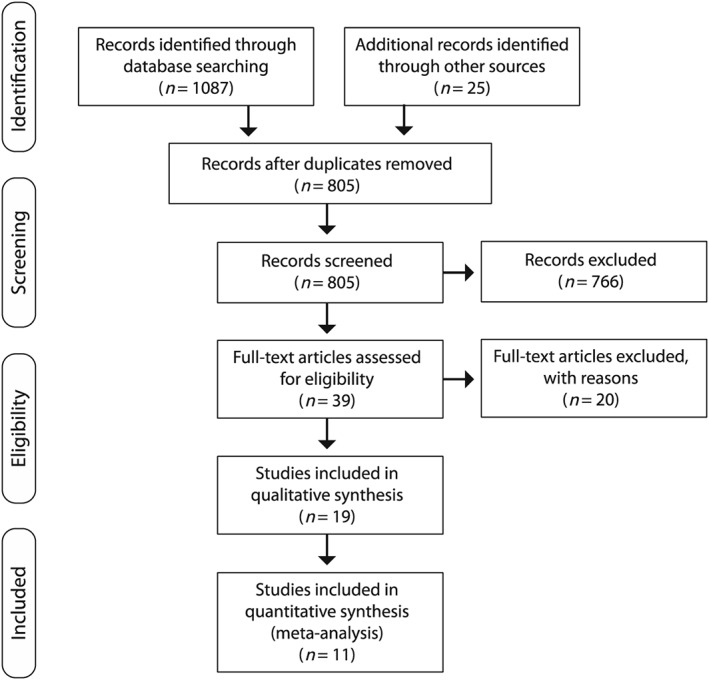
PRISMA (Preferred Reporting Items for Systematic Reviews and Meta‐Analyses) flow diagram; 805 articles were screened by two independent reviewers, and 39 had full‐text assessed. Nineteen articles were included in the review and 11 provided sufficient data to be included in meta‐analysis

The literature search identified 19 studies that could be included in the systematic review 1, 2, 4, 5, 6, 7, 8, 9, 11, 12, 17, 18, 19, 23, 24, 25, 33, 34, 53, and 11 with sufficient data to be included in the meta‐analysis 1, 5, 6, 7, 8, 9, 11, 12. Of the 20 articles excluded during full text screening, 12 studies failed to measure hangover at testing, two of which 30 were reported in the same article 21, 27, 28, 29, 31, 32, 54, 55, 56, 57. Two studies, which included measures on subjective feelings during hangover, only found increases in fatigue or arousal 14, 20, therefore it was unclear if participants were experiencing a hangover. Seven studies failed to measure BAC at testing 3, 10, 27, 29, 30, 54, 58, and two studies which did measure BAC showed that participants achieved BAC > 0.02% 21, 26. Two studies included other treatments in their research design 20, 59. To avoid interference from either the substance or the placebo effect, these studies were excluded. Finally, a further study was excluded 60, as the data analysed were already included in this review via another article from the same authors 9. Figure 1 represents a PRISMA (Preferred Reporting Items for Systematic Reviews and Meta‐Analyses) diagram of study exclusion. Assuming studies which did not report participant attrition did not experience any, total participants recruited across all included studies was 1163. The total number of participants for which data were reported was 846, an attrition rate of 27.3%.

### Included studies

**Figure 2:**
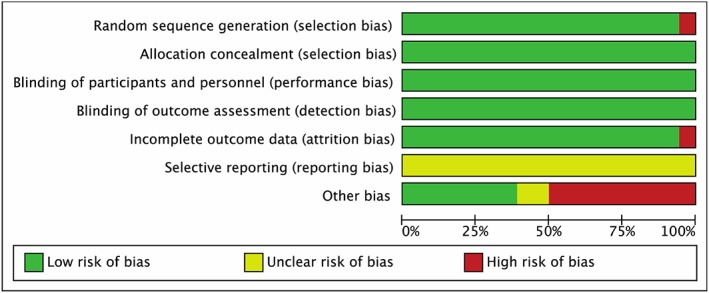
Risk of bias graph. One study was at risk of insufficient randomization procedures; all studies were at risk of reporting bias as there were no pre‐registered study protocols, and 50% of studies were at risk of biases such as non‐randomized task order and sampling bias. [Colour figure can be viewed at http://wileyonlinelibrary.com]

A total of 19 studies were included in the qualitative synthesis, as illustrated in Table 1. The 11 laboratory studies 1, 2, 4, 5, 6, 11, 17, 18, 33, 34, 53 typically administered lower doses of alcohol than were consumed during the eight naturalistic drinking studies 7, 8, 9, 11, 12, 19, 23, 24, 25. Ten studies explored multiple aspects of cognition 1, 2, 5, 7, 8, 9, 11, 18, 19. Risk of bias was assessed using RevMan (56; see Fig. 2). One study did not randomize sufficiently to condition 2, and for all studies it was unclear whether there was bias for selective reporting due to a lack of study pre‐registration. Fifty per cent of studies were at risk of other biases, including non‐randomization of task administration and sampling biases. Blinding was not considered a risk of bias as participants readily guess conditions during experimental hangover research, despite blinding 5, 6.

**Table 1:** Description of included studies.

Study	n	Design	Alcohol	BAC at testing	Hangover measure	Tests used	Cognitive domain	Main finding	Comments
Collins & Chiles, 1979	11	Within‐subjects, laboratory	13 g/kg	< 0.01%	20‐item hangover questionnaire	Choice RT Meter Monitoring Pattern Identification Compensatory Tracking Problem solving	P SA STM DA PS	Non‐significant results	
Collins 1980	8	Within‐subjects, laboratory	1.3 g/kg	0.012%	20‐item hangover questionnaire	Tracking task with RT	DA	Non‐significant results	
Finnigan *et al*. 2005	71	2 × 3 mixed design, naturalistic	1.77 g/kg	0%	Subjective feelings questionnaire	Psychomotor vigilance Dual task Probe memory recall	SA DA STM	Non‐significant results	Group impaired in V, post‐hoc significant for ‘acute and hangover’ only
Grange *et al*. 2016	31	Within‐subjects, naturalistic	1.55 g/kg	0%	AHS	Choice RT	P	Impaired RT	Anecdotal evidence for impaired accuracy
Howland *et al*. 2010	184–193	Within‐subjects, laboratory	0.99 g/kg	0%	AHS	PVT CPT ADST‐B APASAT VST‐B PMT	SA SA WM WM WM STM	Impaired Non‐significant Non‐significant Non‐significant Impaired Female only impairment	
Kim *et al*. 2003	13	Within‐subjects, naturalistic	1.5 g/kg	< 0.01%	Subjective Hangover Scale	LNNB	Various	Impairments in ‘memory’, ‘Visual’ and ‘intellectual’ components	Excluded from meta‐analysis as components cannot be subcategorized
Kruisselbrink *et al*. 2006	12	Within‐subjects, laboratory	1.36 g/kg	0%	Rated common symptoms	Choice RT	P	Non‐significant RT Impaired accuracy	Female participants Alcohol g/kg maximum dose
Laurell & Törnros, 1983	22	Within‐subjects, naturalistic	1.25 g/kg	0	Rated severity	Driving ability	RL	Impaired	
McKinney *et al*. 2004	48	Within‐subjects, naturalistic	1.54 g/kg	< 0.01%	Questionnaire on signs & symptoms	Free recall Delayed recognition Simple RT Choice RT	STM LTM P P	Impaired	STM impaired at 9:00 a.m. only, alcohol g/kg averaged male & female
McKinney *et al*. 2007	78	Mixed design, naturalistic	1.67 g/kg	< 0.01%	Questionnaire on signs & symptoms	Free recall Delayed recognition Simple RT Choice RT	STM LTM P P	Impaired	Stressor between‐subject condition. ES calculated for group effect (hangover/no‐hangover). Alcohol g/kg averaged male & female
McKinney *et al*. 2012	48	Within‐subjects, naturalistic	1.54 g/kg	< 0.01%	Questionnaire on signs & symptoms	Sustained attention Divided attention Erikson Flanker Stroop Spatial attention	SA DA SelA SelA SpaA	Impaired Non‐significant Impaired Impaired Non‐significant	Alcohol g/kg averaged male & female
Mrysten *et al*. 1970	15	Within‐subjects, laboratory	1.43 g/kg	< 0.01%	Rated severity	Simple RT Choice RT *F*‐test Correction test	P P EF SA	All non‐significant except ‘spatial’ factor of *F*‐test	
Rohers *et al*. 1991	5	Within‐subjects, laboratory	0.8 g/kg	0%	Rated hangover	Divided attention	DA	Impaired tracking, but not RT	
Rohsenow *et al*. 2006	61	2 × 2 mixed, laboratory	1.1 g/kg	< 0.02%	AHS	Simulated ship performance	PS	Non‐significant	Outcome overall time. Alcohol g/kg averaged male & female
Rohsenow *et al*. 2010	89–95	2 × 2 × 2 mixed, laboratory	1.15 g/kg	0	AHS	PVT CPT ADST‐B APASAT VST‐B PMT	SA SA WM WM WM STM	Impaired Impaired Non‐significant Non‐significant Non‐significant Non‐significant	Alcohol g/kg averaged male & female
Streufert *et al*. 1995	21	Within‐subjects, laboratory	1 g/kg	0	Drug effects questionnaire	Managerial simulations	EF	Non‐significant	Involved decision making and planning
Törnros & Laurell, 1991	24	Within‐subjects, naturalistic	1.42 g/kg	< 0.02%^a^	Rated severity	Driving speed	RL	Non‐significant	overall impaired, post‐hoc BAC < 0.02% non‐significant
Verster *et al*. 2003	48	Within‐subjects, naturalistic	1.4 g/kg	0	Severity scored	Immediate recall Delayed recall Delayed recognition Macworth clock	STM LTM LTM VA	Non‐significant Impaired Non‐significant Non‐significant	46 participants completed memory tasks
Verster *et al*. 2014	42	Within‐subjects, naturalistic	1.55 g/kg	< 0.01^b^	Severity scored	Driving ability	RL	Ability impaired Speed non‐significant	Alcohol g/kg averaged male & female

P = psychomotor; SA = sustained attention; DA = divided attention; SelA = selective attention; SpaA = spatial attention; VA = vigilance attention; STM = short‐term memory; LTM = long‐term memory; WM = working memory; PS = problem solving; EF = executive function (non‐specified); RL = ‘real‐life’; AHS = acute hangover scale.

a BAC > 0.02% at 9 a.m. session;

b BAC > 0.02% for four participants; however, inclusion did not impact results (correspondence with authors).

### Attention

#### Sustained attention

**Figure 3:**
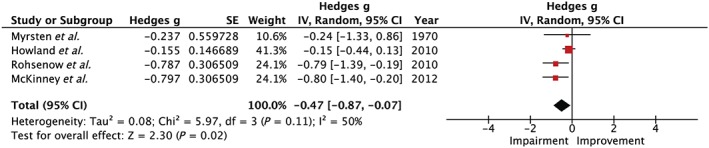
Forest plot for sustained attention. Testing for an overall effect revealed a significant impairment (P = 0.02) with a small to medium effect estimate of 0.47, 95% confidence interval = 0.07–0.87. [Colour figure can be viewed at http://wileyonlinelibrary.com]

Five studies explored sustained attention, three laboratory 1, 5, 6 and two naturalistic 7, 19. Howland *et al*. 6, McKinney *et al*. 7 and Rohsenow *et al*. 5 reported impairments, whereas Finnigan *et al*. 19 and Mrysten *et al*. 1 showed no evidence of next‐day effects on sustained attention. Two studies used tasks from a tool validated for assessing cognitive impairments: the neurobehavioural evaluation system‐3 5, 6, 61, two used a sustained attention task which presented stimuli at a consistent rate and participants responded to consecutive stimuli 7, 19, and one used a ‘correction test’ where participants marked identical rows in a list of two columns 1. Four studies provided sufficient information to be included in the meta‐analysis 1, 7, which revealed an overall impairment in sustained attention during hangover [Hedges’ *g* = 0.47, 95% confidence interval (CI) = 0.07–0.87, *I* ^2^ = 50%]. This is shown graphically in Fig. 3.

#### Divided attention

Five studies included measures of divided attention 4, 7, 17, 18, 19. Of these, one (with a small sample size; *n* = 5) reported impairments in divided attention 4. The four other studies showed no evidence of a next‐day effect on divided attention. Four studies were included in a meta‐analysis 4, 7, 17, 18 which showed no evidence of a next‐day effect on divided attention.

#### Other attention

Verster *et al*. 11 analysed vigilance using the Macworth clock test and found no evidence of next‐day effects. McKinney *et al*. 7 found slowed reaction times (RT) for both near and far distractors in a selective attention task, and increased interference the day after heavy alcohol consumption in the Stroop test. As only one study explored vigilance and only one explored selective attention, a meta‐analysis was not performed for these categories of attention.

### Memory

#### Short‐term memory

**Figure 4:**
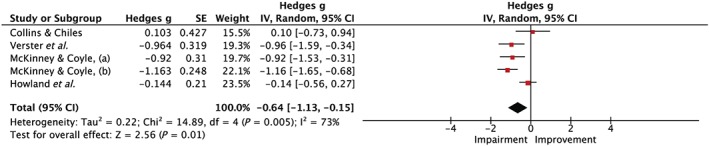
Forest plot for short‐term memory. Testing for an overall effect revealed a significant impairment (P = 0.01) with a medium effect estimate of 0.64, 95% confidence interval = 0.15–1.13. [Colour figure can be viewed at http://wileyonlinelibrary.com]

Short‐term memory (STM) was assessed in seven studies 5, 6, 8, 9, 11, 18, 19, three naturalistic 8, 9, 19 and four laboratory 5, 6, 11, 18. McKinney & Coyle 8, 9 and Howland *et al*. 6 reported impairments, with Howland *et al*. showing a female only impairment, whereas Collins & Chiles 18, Finnigan *et al*. 19, Rohsenow *et al*. 5 and Verster *et al*. 11 reported no evidence of a next‐day effect. Three studies used a word recall task 8, 9, 11, one used a similar task which measured probed recall 19, two used a pattern memory test 5, 6 and one used a ‘pattern identification task’ 18. Five studies provided sufficient information to be included in the meta‐analysis 6, 8, 9, 11, 18 which, as indicated in Fig. 4, revealed an overall impairment for STM during hangover (Hedges’ *g* = 0.64, 95% CI = 0.15–1.13, *I* ^2^ = 73%).

#### Long‐term memory

**Figure 5:**
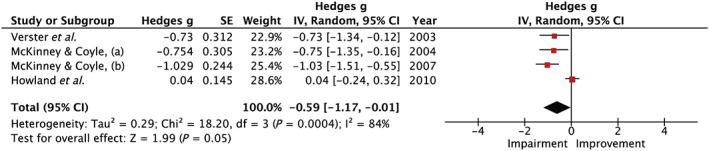
Forest plot for long‐term memory. Testing for an overall effect revealed a significant impairment (P = 0.05) with a medium effect estimate of 0.59, 95% confidence interval = 0.01–1.17. [Colour figure can be viewed at http://wileyonlinelibrary.com]

Four studies, two naturalistic 8, 9 and two laboratory 6, 11, assessed LTM. Verster *et al*. 11 and McKinney & Coyle 8, 9 used a word recall task and reported impairments in LTM. However, in Howland *et al*. 6, where participants were required to learn lecture materials pre‐intoxication, there was no evidence of a next‐day effect on LTM. Figure 5 shows that when all four studies were included in a meta‐analysis there was an overall impairment in LTM during hangover (Hedges’ *g* = 0.59, 95% CI = 0.01–1.17, *I* ^2^ = 84%).

#### Other memory

Howland *et al*. 6 and Rohsenow *et al*. 5 investigated working memory using the adaptive paced auditory serial addition test (APASAT), the visual span‐backwards (VST‐B) and the auditory digit span‐backwards (ADS‐B). They found no evidence of a next‐day effect in the APASAT or the ADS‐B; however, Howland *et al*. 6 reported impairments in the VST‐B during hangover. Kim *et al*. 2 also reported impairments in the memory domain of the Luria‐Nebraska Neurobehavioural Battery (LNNB), although as this domain encompasses STM, LTM and WM 62, it is unclear which aspects of memory were impaired.

### Psychomotor performance

#### Speed

**Figure 6:**
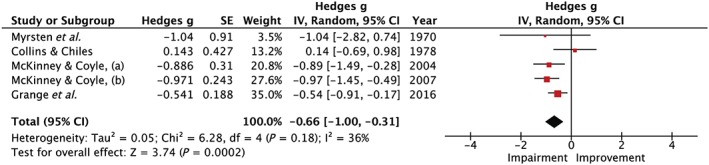
Forest plot for psychomotor speed. Testing for an overall effect revealed a significant impairment (P < 0.001) with a medium effect estimate of 0.66, 95% confidence interval = 0.31–1.00. [Colour figure can be viewed at http://wileyonlinelibrary.com]

Psychomotor speed was measured using RT in six studies 1, 8, 9, 12, 18, 53. Three naturalistic studies 8, 9, 12 found slower RT the day after an evening of heavy alcohol consumption, whereas three laboratory studies found no evidence to support this 1, 18, 53. Five studies 1, 8, 9, 12, 18 were included in the meta‐analysis which, as shown in Fig. 6, indicated that psychomotor speed was slowed the following day (Hedges’ *g* = 0.66, 95% CI = 0.31–1.00, *I* ^2^ = 36%).

#### Accuracy

Two studies reported psychomotor accuracy 12, 53. Kruisselbrink *et al*. 53 found a decrease in psychomotor accuracy following an evening of heavy alcohol consumption, whereas Grange *et al*. 12 reported no evidence of an effect on accuracy.

### ‘Real‐life’ simulations

Six studies included a ‘real‐life’ simulation that required cognitive performance. Rohsenow *et al*. 34 reported no evidence of an effect for solving a mechanical failure during a simulated ship scenario. Streufert *et al*. 33 reported no clear evidence of an effect on performance in scenarios which require managerial skills, and Howland *et al*. 6 reported no evidence of a next‐day effect for General Record Examination scores on two factors; verbal and quantitative. For studies that analysed driving following an evening of heavy alcohol consumption 23, 24, 25, the ability to control a vehicle, as measured by deviation from a set course, was impaired 23, 25, whereas there was no clear evidence to suggest a next‐day effect on driving speed 24. Due to considerable differences in research methodology it was not possible to conduct a meta‐analysis for ‘real‐life’ simulations.

## Discussion

The systematic review and meta‐analyses indicate that STM, LTM sustained attention and psychomotor speed are impaired the day after an evening of heavy alcohol consumption. Results were mixed for the impact of next‐day effects on WM, and there was no clear evidence of an effect on divided attention or vigilance, suggesting that specific components of cognition are influenced the next day. The meta‐analysis showed that psychomotor speed, STM and LTM had medium overall effect estimates (Hedges’ *g =* 0.66, 0.64 and 0.59, respectively), and sustained attention had a small effect estimate (Hedges’ *g =* 0.47).

Our systematic review indicated that sustained attention was impaired in studies using naturalistic and laboratory methodologies, with meta‐analysis revealing an overall impairment with a small effect size (Hedges’ *g* = 0.47). For divided attention, only Rohers *et al*. reported an impairment 4; however, the reliability of this study is potentially limited by the small sample size (*n* = 5). Meta‐analysis data revealed no evidence of a next‐day effect on divided attention. Next‐day impairments in sustained attention may reflect accumulating mental fatigue, induced by prolonged attentional demands 63. Fatigue is a common symptom of hangover 64 and involves reward‐cost trade‐offs 65, 66. Therefore, hangover‐induced fatigue may contribute to impairments observed in sustained attention. The lack of clear evidence for an effect in some studies of sustained attention may reflect insensitivity of the cognitive task used. Studies which used tasks that have not previously demonstrated sensitivity to state changes in drug use 1, 17, 18 tended to report no evidence of a next‐day effect, whereas studies 7 that used cognitive tasks that have previously detected state changes 67 were more likely to report next‐day related impairments. Next‐day effects on sustained and divided attention may also have been masked by low statistical power. For example, Finnigan *et al*. 19 had small, unequal group sizes in their between‐subjects design (*n* = 13, *n* = 25, *n* = 33 for ‘acute and hangover’, hangover and control groups, respectively).

Our review highlights converging evidence from both methodologies (experimental and naturalistic) that STM and LTM may be influenced the morning following a night of heavy alcohol consumption, with the meta‐analysis revealing impairments in both. It is possible that memory formation, rather than retrieval, may be affected, as indicated by the differential next‐day effects on studies in which learning took place following heavy alcohol consumption versus sober state. An important process for memory formation in the hippocampus is long‐term potentiation (LTP)—the strengthening of signals between neurones 68. Given the detrimental effect of elevated interleukin (IL)‐6 69, 70, 71 and cortisol 72 on LTP 73 and the increase of these in the morning following heavy alcohol consumption 74, 75, 76, this could be a possible mechanism underlying next‐day related impairment of memory formation. Three studies examined memory processes using a naturalistic methodology, two of which reported impairments in STM and LTM, whereas Finnigan *et al*. 19 reported no evidence of impairments in STM. However, as mentioned above, this study may have lacked the statistical power to identify next‐day effects. Conversely, experimental studies have largely reported no evidence of next‐day impairment of memory, although studies where participants reached higher BACs tended to report impairments 6, 11. As with studies of attention, some studies that reported no clear evidence of an effect on memory may have used tasks that are insensitive to the acute next‐day effects.

Systematic review revealed conflicting results for next‐day influences on psychomotor speed. However, when effect estimates were combined in the meta‐analysis, there was an overall impairment with a medium effect estimate (Hedges’ *g* = 0.66). It is important to consider the suitability of RT as an outcome measure when assessing the next‐day effects on cognition. For example, Howland *et al*. 6 and Rohsenow *et al*. 5 use RT as an outcome measure in tasks of sustained attention. Both reveal impairments; however, it is unclear whether the impairment is related to sustained attention or psychomotor speed. Some cognitive tasks of sustained attention, which do not use RT as an outcome measure, revealed no clear evidence of next‐day effects on attention 1. Three naturalistic studies reported slower RTs, whereas three laboratory studies reported no evidence of an effect, although Kruisselbrink *et al*. 53 reported decreased accuracy. Studies using experimental manipulation of ‘hangover’ typically administered lower doses of alcohol than studies where ‘hangover’ occurred ‘naturally’ (1.3–1.43 and ~1.54–1.67 g/kg, respectively), and had smaller sample sizes (*n* = 8–12), which may impact reliability 77. It should be noted that, due to insufficient information, one laboratory study 53 could not be included in the meta‐analysis, which may over‐inflate the effect estimate reported.

Three naturalistic studies identified in this review assessed driving the morning following a night of heavy alcohol consumption. Verster *et al*. and Laurell & Törnros 23, 25 reported impairments in ability to control the vehicle. However, Törnros & Laurell 24 reported no effect on speed the next day. These studies have important implications for road safety, especially given that hangover may contribute to road‐traffic accidents 78. The impairments observed in ability to drive may be driven by next‐day effects on underlying cognitive components. Driving uses psychomotor speed and sustained attention 79, both of which appear to be impaired in this review. Studies using experimental manipulation of hangover, which assessed task performance using measures of executive function (problem‐solving and decision‐making), as well as academic performance, all found no clear evidence of a next‐day effect. However, an outcome measure of overall completion time, as in Rohsenow *et al*. 34, and the managerial task used in Streufert *et al*. 33, may not be sufficient to detect next‐day effects. Together, these findings echo the recommendations of previous reviews 36, 41, indicating that further research is needed to determine hangover effects on executive functions. We also suggest that future studies of executive function should use validated measures known to be sensitive to state changes in drug use, such as the Iowa Gambling Task 80.

In line with previous reviews 35, 36, 41, 46, this systematic review and meta‐analysis revealed several methodological issues which limit the interpretation of evidence from studies of alcohol hangover on cognition. Although the studies included in this review met rigorous criteria there was a high degree of variability in the design of individual studies, possibly reflected by the high level of heterogeneity observed. Our review highlights that low sensitivity of tasks to detect next‐day impairments may underlie null next‐day effects on cognition. The use of cognitive tasks sensitive to state changes in substance use is essential for studies exploring cognitive effects the day after a night of heavy alcohol consumption 81. Thought should also be given to the sensitivity of visual stimuli to next‐day effects, as opposed to auditory stimuli. Studies using cognitive tasks with auditory stimuli revealed no evidence of a next‐day effect on cognition, in contrast to effects observed when using visual stimuli. This discrepancy is supported by evidence of impairments of the ‘visual’ component of the LNNB task battery 2. Another factor that may influence the next‐day effect on cognition is study design. Our review suggests a greater likelihood of next‐day impairment in studies of naturalistic design. In studies where hangover is induced ‘naturally’, alcohol consumption was higher (mean alcohol dose = 1.54 g/kg) than in experimental studies (mean alcohol dose = 1.21 g/kg). This finding suggests that higher alcohol doses are associated with greater next‐day performance impairments 82.

Finally, several other limitations should be considered. One study 2 did not randomize condition order, while others did not randomize task administration order 7, 8, 9. Randomization to condition is important to prevent practice effects, and randomizing task order limits confounding variables such as fatigue. Several studies did not control for nicotine use 1, 17, 18, which is known to influence cognitive performance 83. Our review also highlighted variability between study design in the amount of time between alcohol consumption and cognitive testing, possibly depriving participants of sleep. Sleep time is an important consideration when researching cognition, as cognitive components are affected differentially by sleep loss 84. Although in real‐life drinking some individuals may reduce sleep time for drinking time 85, variability between studies for the time allowed for sleep make it difficult to draw firm conclusions regarding cognitive effects.

Based on these shortcomings, we make the following recommendations for future research. First, to address the shortcoming of low statistical power, studies should conduct a priori power analysis to determine an estimate of required sample sizes. Secondly, studies should adopt tasks that have been validated and shown to be sensitive to state changes in drug use. Thirdly, consideration should be given for the use of RT as an outcome measure in tasks, and interpretation should acknowledge the potential impact of next‐day psychomotor impairments. Fourthly, future research should seek to address the paucity of robust research examining executive functions the morning following a night of heavy alcohol consumption.

## Conclusion

To our knowledge, this study is the first to systematically review the literature exploring next‐day effects on cognitive performance and to estimate mean effect sizes. Our review reveals next‐day impairments in STM, LTM, psychomotor speed and sustained attention, with mixed findings for next‐day effects on working memory, and no clear evidence of an effect on divided attention. Results from our meta‐analysis indicate medium effect sizes for psychomotor speed, STM and LTM, and a small effect size for sustained attention. These findings suggest that specific cognitive functions may be impaired the morning following a night of heavy alcohol consumption, with implications for everyday task performance (e.g. driving).

## Declaration of interests

S.A. and M.R.M. are members of the UK Centre for Tobacco and Alcohol Studies. The other authors have no relevant conflicts of interest to disclose.
